# Endometrial Regenerative Cell-Derived Exosomes Attenuate Experimental Colitis through Downregulation of Intestine Ferroptosis

**DOI:** 10.1155/2022/3014123

**Published:** 2022-08-22

**Authors:** Yanglin Zhu, Hong Qin, Chenglu Sun, Bo Shao, Guangming Li, Yafei Qin, Dejun Kong, Shaohua Ren, Hongda Wang, Zhaobo Wang, Jingyi Zhang, Hao Wang

**Affiliations:** ^1^Department of General Surgery, Tianjin Medical University General Hospital, Tianjin 300052, China; ^2^Tianjin General Surgery Institute, Tianjin Medical University General Hospital, Tianjin 300052, China; ^3^Nankai University, School of Medicine, Tianjin 300071, China; ^4^School of Basic Medical Sciences, Tianjin Medical University, Tianjin 300052, China

## Abstract

**Background:**

Endometrial regenerative cells (ERCs) have been identified to ameliorate colitis in mice; however, whether exosomes derived from ERCs (ERC-exos) own similar effects on colitis remains unclear. Ferroptosis, an iron-dependent cell programmed death form, has been reported to promote inflammation in UC. Thus, in this study, whether ERC-exos can treat colitis and regulate intestine ferroptosis will be explored.

**Methods:**

In this study, iron, malondialdehyde (MDA) production, glutathione (GSH) synthesis, and acyl-CoA synthetase long-chain family member (ACSL) 4 and glutathione peroxidase 4 (GPX4) expressions were measured in colon samples from healthy people and UC patients to explore the effects of ferroptosis. *In vitro*, ERC-exos were cocultured with ferroptosis inducer erastin-treated NCM460 human intestinal epithelial cell line, and ferroptotic parameters were measured. *In vivo*, colitis was induced by 3% dextran sulfate sodium (DSS) in BALB/c mice, and animals were randomly assigned to normal, untreated, and ERC-exos-treated groups. The Disease Activity Index (DAI) score, histological features, tissue iron, MDA, GSH, ACSL4, and GPX4 were measured to verify the role of ERC-exos in attenuating UC.

**Results:**

Compared with healthy people, UC samples exhibited higher levels of iron, MDA, and ACSL4, while less levels of GSH and GPX4. *In vitro*, the CCK-8 assay showed that ERC-exos rescued erastin-induced cell death, and ERC-exos treatment significantly increased the levels of GSH and expression of GPX4, while markedly decreasing the levels of iron, MDA, and expression of ACSL4. *In vivo*, ERC-exos treatment effectively reduced DAI score, ameliorated colon pathological damage, and improved disease symptoms. Moreover, ERC-exos treatment further enhanced the levels of GSH and the expression of GPX4 but reduced the levels of iron, MDA, and expression of ACSL4 in the colon of colitis mice.

**Conclusions:**

Ferroptosis was involved in the pathogenesis of UC, and ERC-exos attenuated DSS-induced colitis through downregulating intestine ferroptosis. This study may provide a novel insight into treating UC in the future.

## 1. Introduction

Ulcerative colitis (UC) is a chronic nonspecific inflammatory disease, which mainly affects the colon and rectum [1]. Occurring with rapid socioeconomic development, the prevalence of UC is increasing which continues to challenge the healthcare system all over the world [2]. At present, the pathogenesis of UC may involve imbalanced immune homeostasis, disordered intestinal flora, and inappropriate survival environment [3–5]. Although drugs for UC are various including hormones [6], biological agents [7], and immunosuppressants [8], during the application of these drugs, side effects such as renal impairment [9], severe infection [10], and venous thromboembolism [11] cannot be ignored. Hence, exploring novel available strategies for treating UC is urgently required.

It has been proved that based on mesenchymal stem cell- (MSC-) mediated therapy on UC is effective and promising. Endometrial regenerative cells (ERCs) are a novel source of adult stem cells [12], which are collected from menstrual blood and own similar properties to MSCs [13]. Surprisingly, compared with MSCs, ERCs have unique advantages of abundant sources, noninvasive acquisition [14, 15], and so on. In addition, studies showed that ERCs amplifies twice as fast as bone marrow-MSCs (BM-MSCs) [14] and the yield is 2-4-fold higher than that of BM-MSCs [16]. ERCs have been identified to exert therapeutic effects on experimental colitis [17], acute immune-mediated hepatitis [18], myocardial injury [19], and cardiac allograft rejection [20], etc. by our team and others. As known, the way MSCs exert therapeutic effects is mainly through paracrine mechanism, including cytokines, free mitochondria, and exosomes [21]. Exosomes are biological nanovesicles with a diameter of 30-150 nm containing diverse biomolecules, such as lipids, proteins, and nucleic acids [22], which can function as a unidirectional carrier to transfer signals among cells [23]. Superior to MSCs, MSC-derived exosomes (MSC-exos) have higher chemical stability, longer distance intercellular communication, and stronger autonomous targeting capabilities [24]. Previous studies have demonstrated that MSC-exos can attenuate experimental colitis *via* regulating immune cells [25]. Our previous study has revealed ERC-derived condition medium can treat colitis, which may involve ERC-exos' participation [26].

Ferroptosis is an iron-dependent programmed cell death, which was coined fairly in 2012 [27]. In 2018, the Nomenclature Committee on Cell Death defined ferroptosis as a form of regulated cell death initiated by intracellular oxidative perturbations, which is under constitutive control by GPX4 and can be inhibited by iron chelators and lipophilic antioxidants [28]. Studies have verified that ferroptosis was involved in the pathogenesis of various disease, including cancers [29], ischemia/reperfusion injuries (IRI) [30], cardiovascular disease [31], stroke [32], and inflammatory diseases [33]. Xu et al. have revealed that ferroptosis participated in UC [34] and Dong et al. and Wang et al. have demonstrated that inhibiting ferroptosis could alleviate UC [35, 36]. Therefore, focusing on regulating ferroptosis may provide a novel insight into the treatment of UC.

Given the promising therapeutic effects of ERC-exos and inspired by previous studies of ferroptosis, the current study was designed to investigate whether ERC-exos can attenuate experimental colitis through regulating intestine ferroptosis.

## 2. Materials and Methods

### 2.1. Clinical Samples

Colon samples of UC patients (*n* = 10) and healthy control (*n* = 10) were collected from the Department of General Surgery, Tianjin Medical University General Hospital. Control colon samples were taken from normal intestinal tissue removed during surgery in patients with colorectal cancer. All samples used in this study were approved by the Ethical Committee of Tianjin Medical University General Hospital (Ethic No. IRB2022-WZ-031). Samples soaked in formalin were used to make paraffin sections for detecting the expression of GPX4, and frozen tissues were used to assess the levels of iron, GSH, and MDA. In addition, proteins extracted from samples were used to measure the expressions of GPX4 and ACSL4.

### 2.2. Animals

Male BALB/c mice, aging 6-8 weeks and weighing 22-25 g, were purchased from China Food and Drug Inspection Institute (Beijing, China). All animals were housed in a conventional experimental environment with enough food and water following the guidelines of the China Association for the Production of Animals and the protocol was approved by the Animal Ethical and Welfare Committee of Tianjin Medical University General Hospital (ethic no. IRB2022-DW-07).

### 2.3. Experimental Groups

Colitis was induced by feeding with water containing 3% DSS (Yeasen, Shanghai, China). All animals were randomly assigned to three groups (*n* = 6 per group): the normal control group, untreated group, and ERC-exos-treated group. The mice in normal control group were fed with free water for 10 days while the other two groups were fed with water containing 3% DSS for 7 days and then administered water for 3 days as described previously [17]. ERC-exos-treated mice were injected with ERC-exos (100 *μ*g total protein in 200 *μ*L volume) *via* the tail veins on days 2, 5, and 8 of DSS administration. On day 10, mice were killed for the collection of colon samples which were excised from the ileocecal junction to the anus.

### 2.4. Preparations of ERCs and ERC-exos

The menstrual blood of healthy woman volunteers were collected on the second day of the menstrual cycle (20-30 years old). ERC harvest and culture were followed as described in the previous study [37]. In brief, the mononuclear cells were isolated via a standard Ficoll method and then were cultured in Dulbecco's modified Eagle's medium supplemented with 10% fetal bovine serum and 1% penicillin/streptomycin. At passage 3, the morphology and the surface markers CD105, CD44, CD45, CD73, CD79a, and HLA-DR of ERCs were examined by microscopy and flow cytometry.

When ERCs reached 60-70% confluency, aspirating the medium and rinsing ERCs three times with sterile PBS were done. After adding a fresh serum-free culture medium (Umibio, Shanghai, China) to incubate cells for 48 h, the conditioned medium were collected and then acquired exosomes by ultracentrifugation. Briefly, the conditioned medium were centrifuged for 10 min at 300 g, 20 min at 2000 g, and 30 min at 10,000 g to remove cell debris. Then, the supernatant was transferred to fresh tubes and centrifuged for 70 min at 110,000 g twice to harvest pure ERC-exos.

### 2.5. Exosome Identification

The exosome from ERC supernatants were verified by nanoparticle, morphology, and surface markers. The size distribution of the precipitated particles was measured by nanoparticle tracking analysis. The size of the particles was analyzed using Zetasizer Software. Results are presented in a particle size distribution graph. The morphology of exosomes was observed by transmission electron microscopy. Briefly, isolated exosomes were resuspended in PBS, and then, exosomes were suspended in 2% glutaraldehyde and loaded onto copper grids, after which they were negatively stained with 3% (*w*/*v*) aqueous phosphotungstic acid for 1 min. Finally, exosomes were observed by transmission electron microscope. ERC-exos protein concentration was determined using a BCA kit (Solarbio, Beijing, China). CD9, CD63, and Alix were used as markers of exosomes in immunoblot assays.

### 2.6. Cell Lines and Treatment

The human normal colonic epithelial cell line NCM460 were used *in vitro*. In the erastin-treated group, ferroptosis inducer 20 *μ*M erastin (MCE, HY-15763) was added to the culture medium after culturing NCM460 for 24 h. And in the ERC-exos-treated group, 20 *μ*M erastin and 15 *μ*g ERC-exos were added to NCM460 at the same time and cocultured for 24 h.

### 2.7. Cell Viability

Cell Counting Kit-8 reagent (CCK-8, Solarbio, Beijing, China) was used to determine the cell viability according to the manufacturer's instructions. Briefly, NCM460 were seeded onto a 96-well plate at a density of 5 × 10^3^ cells/well and treated with vehicle control (DMSO solvent), erastin, and erastin plus ERC-exos. 10 *μ*L CCK-8 was added per well and cultured for 1 h. The absorbance at 450 nm was measured in a multimode microplate reader for cell viability assessment.

### 2.8. Iron, GSH, and MDA Analyses

Protein samples were collected from colon tissues or NCM460 cells to measure the levels of iron, GSH, and MDA. An iron assay kit (TC1015, Leagene, Beijing, China), a GSH assay kit (BC1175, Solarbio, Beijing, China), and an MDA assay kit (A020-2, Jian Cheng, Nanjing, China) were used to determine the levels of iron, GSH, and MDA.

### 2.9. Histology and Immunohistochemistry

All colon samples were fixed in 10% formalin, embedded in paraffin, and then sectioned into 5 *μ*m slices. Hematoxylin and eosin staining was used for examination of histopathological changes. To evaluate ferroptosis in colitis, immunohistochemistry was performed for GPX4. After deparaffinization and rehydration, sections were incubated with 3% H_2_O_2_ to eliminate endogenous peroxides and then heated in the microwave for antigen retrieval. 10% goat serum was used to block nonspecific antigens and sections were finally incubated with rabbit anti-mouse GPX4 antibody (Abcam, ab125066) overnight at 4°C. The next day, horseradish peroxidase-conjugated avidin and brown-colored 3,3′-diaminobenzidine were used to develop the signal and the sections were stained with hematoxylin and then were recorded for analysis.

### 2.10. Western Blot Analysis

Proteins from cell samples and colon tissues were extracted using RIPA lysis mixed with PMSF (Solarbio, Beijing, China) and quantified using a BCA assay kit (Solarbio, Beijing, China). Then, the total protein was separated by sodium dodecyl sulfate polyacrylamide gel electrophoresis and transferred to PVDF membranes. After blocking with 5% skim milk, PVDF membranes were incubated with anti-GPX4 antibody (dilution at 1 : 1000, Abcam, Cambridge, UK), anti-ACSL4 antibody (dilution at 1 : 1000, Abclonal, Wuhan, China), and anti-GAPDH antibody (dilution at 1 : 1000, Sevicebio, Wuhan, China) at 4°C overnight. On the other day, the membranes were incubated with anti-mouse secondary antibody (dilution at 1 : 1000, Servicebio, Wuhan, China) or anti-rabbit secondary antibody (dilution at 1 : 1000, CST, Boston, USA) for 50 min at room temperature. Finally, signals were detected with electrochemiluminescence solution (ECL, Millipore, Massachusetts, USA) using a ChemiScope exposure machine (Clinx Science Instruments Co., Ltd., Shanghai, China) and images were analyzed by ImageJ software.

### 2.11. Statistical Analysis

All data were expressed as mean ± standard deviation (SD). The differences among groups were analyzed by using unpaired *t*-test (groups = 2) or ANOVA analysis (groups ≥ 3). *p* value < 0.05 was considered statistically significant.

## 3. Results

### 3.1. Ferroptosis Was Involved in the Pathogenesis of UC Patients

**Figure 1:**
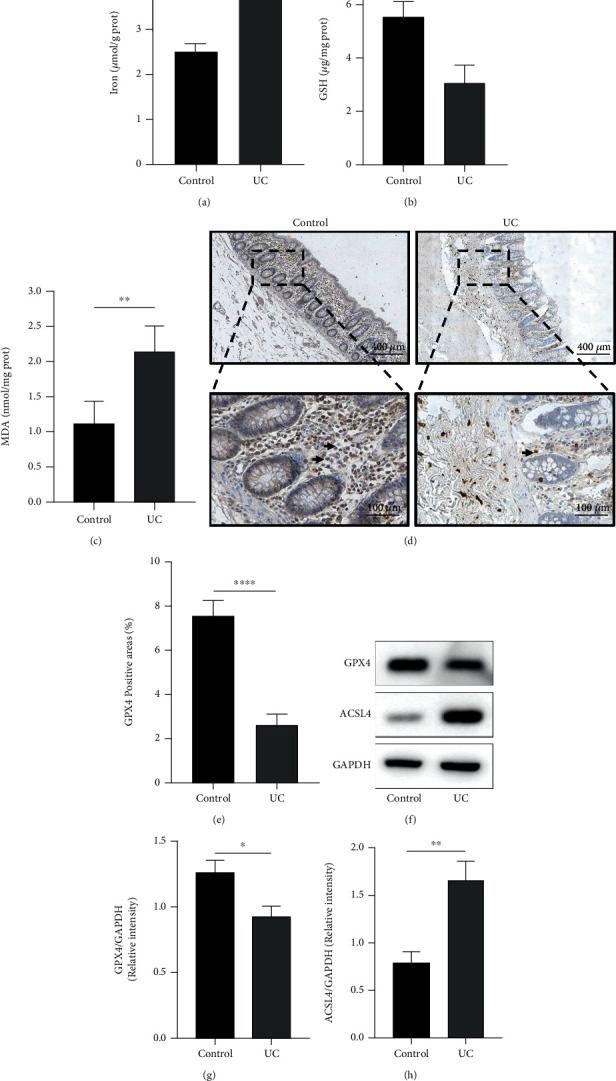
Ferroptosis was involved in the pathogenesis of UC. (a) Iron, (b) GSH levels, and (c) MDA levels were detected in colon samples from the healthy people and UC patients, respectively. (d) Representative images of immunohistochemical analysis measuring GPX4 levels in human colon samples, and (e) quantitative data of cell counts are shown. (f–h) Western blotting analysis of GPX4 and ACSL4. GAPDH was used as the loading control. The *p* value was determined by unpaired *t*-test. ^∗^*p* < 0.05, ^∗∗^*p* < 0.01, ^∗∗∗^*p* < 0.001, and ^∗∗∗∗^*p* < 0.0001.

To verify the presence of ferroptosis in the colon of UC patients, samples were collected from ten UC patients and ten healthy people, respectively, for the measurement. The results showed that the iron was significantly increased in UC compared with the healthy control (Figure 1(a), the control group vs. UC group, *p* < 0.01). The lipid peroxidation product MDA was increased (Figure 1(c), the control group vs. UC group, *p* ≤ 0.01), while GSH synthesis was markedly inhibited in colitis samples (Figure 1(b), the control group vs. UC group, *p* ≤ 0.001). In addition, the levels of GPX4 which directly reduces peroxidized phospholipids as an antioxidant enzyme in colonic specimens were measured by immunohistochemistry and western blot, and results showed a significantly lower expression of GPX4 in UC patients (IHC: Figures 1(d) and 1(e), the control group vs. UC group, *p* < 0.0001; western blot: Figures 1(f) and 1(g), the control group vs. UC group, *p* < 0.05). ACSL4 expression was also increased in the UC patients (Figures 1(f) and 1(h), the control group *vs.* UC group, *p* < 0.01). All these results indicated that ferroptosis was involved in the UC.

### 3.2. Characterization of ERCs and ERC-exos

**Figure 2:**
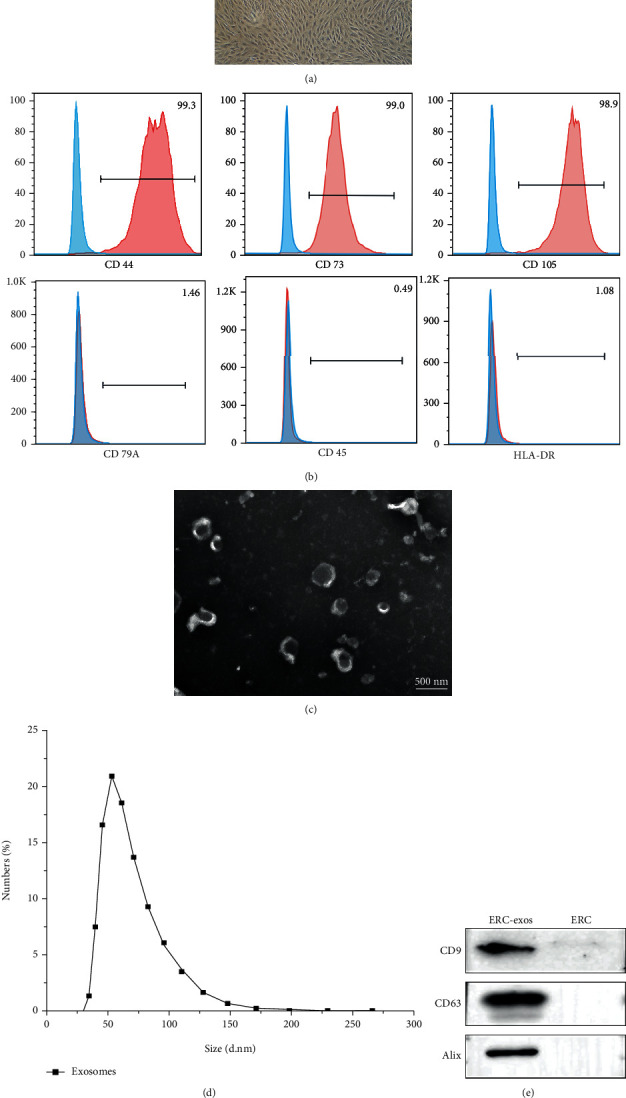
Identification of ERCs and ERC-exos. (a) Morphology of ERCs at P3. (b) Flow cytometry analysis of surface markers of ERCs. (c) Representative image of exosomes. (d) Particle size distribution of exosomes. (e) Immunoblot of exosome markers (CD9, CD63, and Alix).

As shown in Figure 2(a), ERCs exhibited spindle-shaped and fibroblast-like morphology. Flow cytometry analysis showed that ERCs positively expressed CD44, CD105, and CD73, and negatively expressed CD45, CD79a, and HLA-DR (Figure 2(b)). Exosomes from the conditioned medium derived from ERCs exhibited typical lipid bilayer membrane encapsulation (Figure 2(c)) and the particle size distribution ranged from 30 to 150 nm (Figure 2(d)). In addition, the western blot showed that CD63, CD9, and Alix were expressed on exosomes (Figure 2(e)). Taken together, the properties of ERC-exos in this study met the typical criteria for exosomes.

### 3.3. ERC-exos Downregulated NCM460 Ferroptosis *In Vitro*

**Figure 3:**
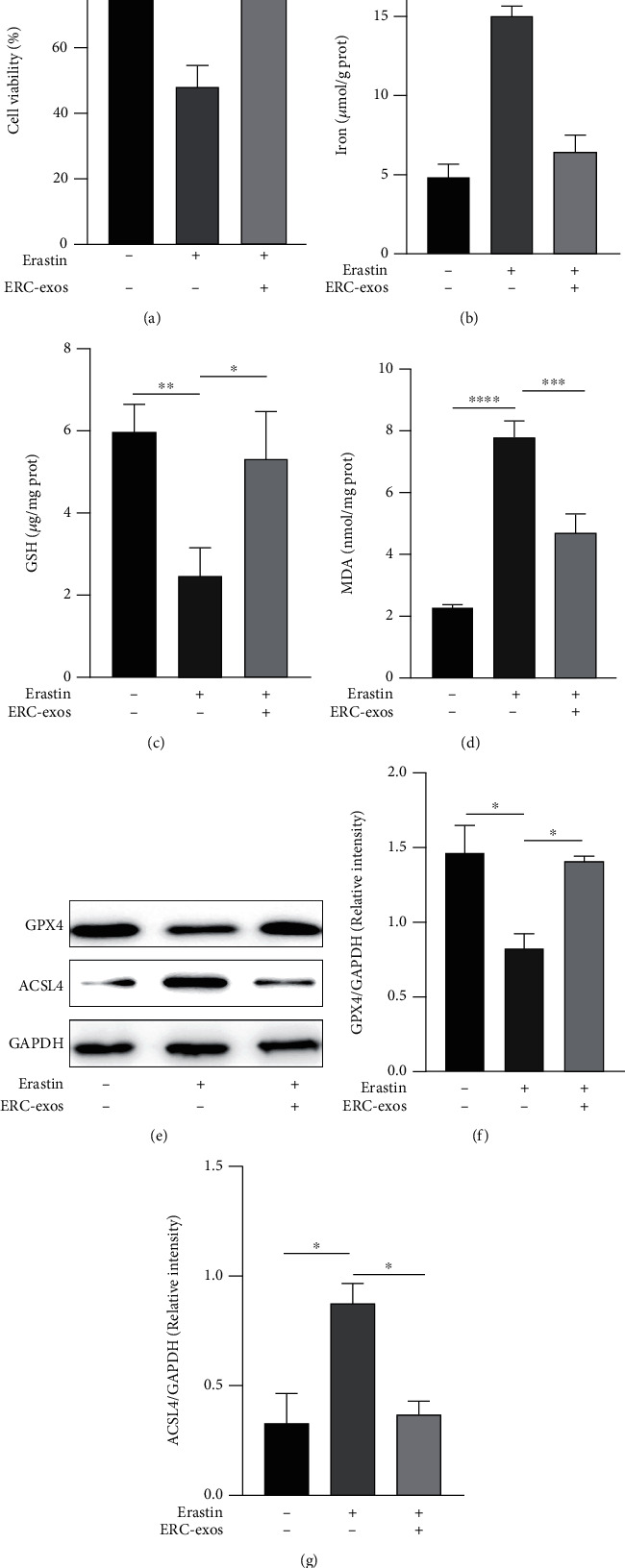
ERC-exos downregulated ferroptosis induced by erastin in NCM460. (a) Relative vitality in erastin-induced NCM460 was measured by CCK-8 kit. The levels of (b) iron, (c) GSH, and (d) MDA in NCM460 treated with erastin or ERC-exos. (e–g) The GPX4 and ACSL4 protein expressions in NCM460 measured by western blot. The *p* value was calculated by one-way ANOVA. ^∗^*p* < 0.05, ^∗∗^*p* < 0.01, ^∗∗∗^*p* < 0.001, and ^∗∗∗∗^*p* < 0.0001.

In order to investigate whether ERC-exos could regulate ferroptosis in the UC, *in vitro* NCM460 cell line was used to mimic the intestine. Erastin was added to culture systems prior to ERC-exos. Cell viability assay revealed that erastin reduced cell viability (Figure 3(a), the erastin group vs. control group, *p* < 0.0001), while ERC-exos obviously increased cell viability (Figure 3(a), the ERC-exos group vs. erastin group, *p* < 0.0001). In addition, cells induced by erastin had higher levels of iron and MDA (Figures 3(b) and 3(d), the iron, erastin group vs. control group, *p* < 0.0001; the MDA, erastin group vs. control group, *p* < 0.0001), as well as inhibiting GSH synthesis (Figure 3(c), the erastin group vs. control group, *p* < 0.01), while the treatment of ERC-exos reversed ferroptosis in cells (Figures 3(b)–3(d),the iron : ERC-exos group vs. erastin group, *p* < 0.0001; the GSH : ERC-exos group vs. erastin group, *p* < 0.05; the MDA : ERC-exos group vs. erastin group, *p* < 0.001). Moreover, treatment of ERC-exos led to decreased level of ACSL4 but an increased level of GPX4 (Figures 3(e)–3(g), the GPX4 : ERC-exos group vs. erastin group, *p* < 0.05; ACSL4 : ERC-exos group vs. erastin group, *p* < 0.05). Collectively, these data suggested that ERC-exos downregulated ferroptosis in erastin-treated NCM460 *in vitro.*

### 3.4. ERC-exos Alleviated DSS-Induced Colitis Symptoms in Mice

**Figure 4:**
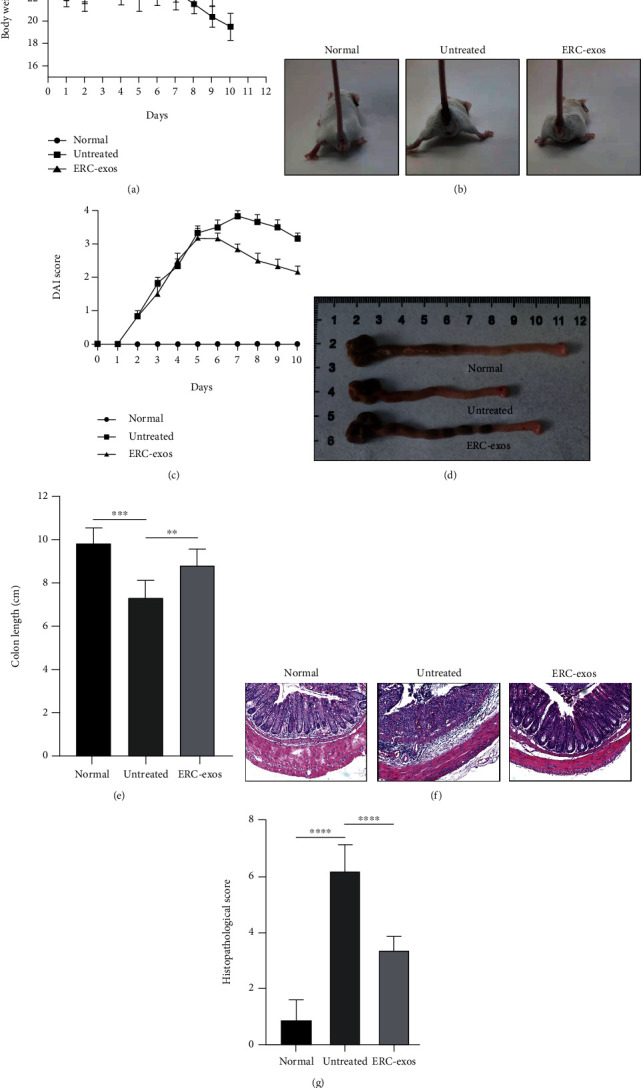
ERC-exos attenuated experimental colitis in mice. (a) Body weight changes of mice from each group. (b) Representative pictures showing bloody stool indifferent groups. (c) DAI score was used to assess the colitis activity. (d, e) The length of colons was measured and analyzed. (f) Representative images of colon tissues in each group (H&E staining). (g) Histopathology scores were evaluated and calculated. The *p* value was determined by one-way ANOVA. ^∗^*p* < 0.05, ^∗∗^*p* < 0.01, ^∗∗∗^*p* < 0.001, and ^∗∗∗∗^*p* < 0.0001.

To further confirm the effects of ERC-exos in colitis, after 3% DSS induction for seven days, the clinical symptoms were evaluated. Mice in the untreated group exhibited colitis with severe bloody stool and weight loss, while ERC-exos-treated mice showed improved symptoms (Figures 4(a) and 4(b)). The Disease Activity Index (DAI) score further reflected the therapeutic effects of ERC-exos (Figure 4(c)). In addition, the shortened colon caused by continuous DSS administration was improved by ERC-exos treatment (Figures 4(d) and 4(e), the untreated group vs. control group, *p* < 0.001; the ERC-exos group *vs.* untreated group, *p* < 0.01). Colon slices from the untreated group showed massive inflammatory cell infiltration, goblet cell depletion, and damaged crypt structure and epithelium cells, whereas ERC-exos ameliorated the pathological changes (Figures 4(f) and 4(g), the untreated group vs. control group, *p* < 0.0001; the ERC-exos group vs. untreated group, *p* < 0.0001). Through the above results, it has been confirmed that ERC-exos relieved experimental colitis.

### 3.5. ERC-exos Could Regulate Ferroptosis in DSS-Induced Colitis

**Figure 5:**
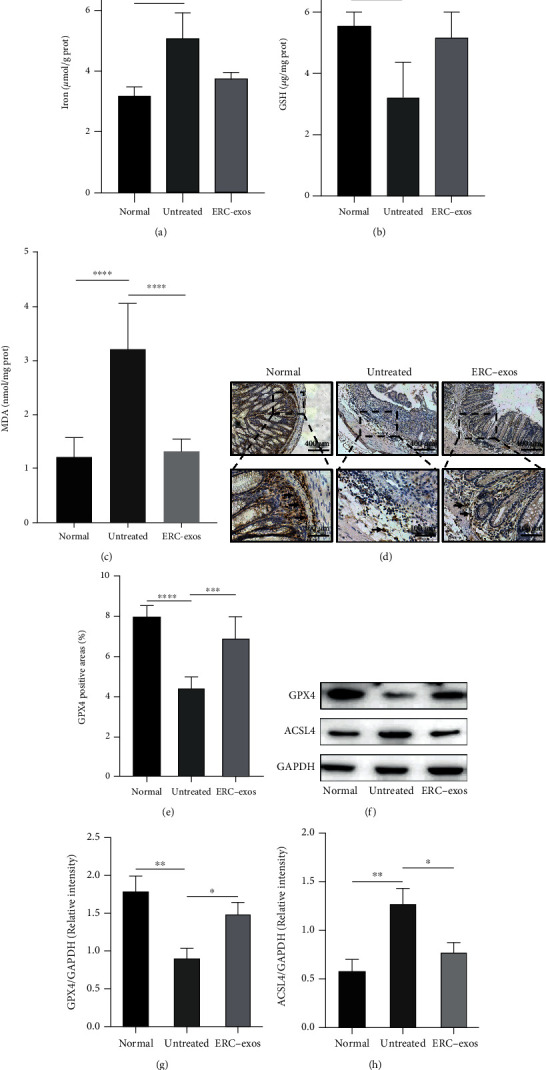
ERC-exos downregulated ferroptosis in mouse colitis model. The levels of (a) iron, (b) GSH, and (c) MDA of colon samples from different groups were measured. (d, e) Representative images of immunohistochemical analysis measuring GPX4 levels in colons from mice. (f–h) Western blot analysis measuring GPX4 and ACSL4 expression levels in mouse colon samples from different groups. GAPDH was used as the loading control. The *p* value was counted by one-way ANOVA. ^∗^*p* < 0.05, ^∗∗^*p* < 0.01, ^∗∗∗^*p* < 0.001, and ^∗∗∗∗^*p* < 0.0001.

To thoroughly verify whether ERC-exos could alleviate colitis through regulation of ferroptosis, iron, GSH, MDA, GPX4, and ACSL4 were measured. Evidence demonstrated that ERC-exos could decrease levels of iron and MDA but increase GSH synthesis (Figures 5(a)–5(c), the iron : ERC-exos group vs. untreated group, *P* < 0.01; the GSH : ERC-exos group vs. untreated group, *p* < 0.01; the MDA : ERC-exos group vs. untreated group, *p* < 0.0001). Immunohistochemistry analysis and western blot further confirmed that ERC-exos effectively promote the GPX4 expression (Figures 5(d)–5(g), the IHC : ERC-exos group vs. untreated group, *p* < 0.001; the WB : ERC-exos group vs. untreated group, *p* < 0.05). In addition, ERC-exos treatment decreased ACSL4 expression in colons (Figures 5(f) and 5(h), the ERC-exos group vs. untreated group, *p* < 0.05). Taken together, these results indicated that ERC-exos could alleviate colitis through downregulating ferroptosis.

## 4. Discussion

UC is a complex disease in which the interaction of genetic, environmental, psychiatric factors, and microbial factors drive chronic intestinal inflammation. In this study, indicators related to ferroptosis were detected in colons from healthy people and UC patients and found that there was a significant increase in ferroptotic parameters in UC patients. *In vitro*, erastin was used to induced ferroptosis in NCM460, whereas ERC-exos treatment markedly downregulated ferroptosis in this cell line. *In vivo*, DSS-induced mice were treated with ERC-exos *via* tail vein injection. Symptoms such as colon length, bloody stool, and weight loss were significantly attenuated in the ERC-exos-treated group. Intestinal pathological results showed that colons in the ERC-exos-treated group presented an improved structure of epithelium and crypts, with abundant goblet cells. All results suggested that ERC-exos attenuated experimental colitis in mice. In addition, by measuring ferroptosis-related indicators we confirmed that ERC-exos downregulate intestine ferroptosis.

Iron is an essential element of the body and a component of many enzymes and immune system compounds; however, it can produce harmful oxygen radicals through the Fenton reaction leading to increased tissue damage and inflammation [38]. One of the pathogenesis of colitis is iron-derived oxidant activity [39]. It has been reported that high dietary iron intake increased the risk of UC [40], iron overload led to dysregulated reactive oxygen species (ROS) generation, and interference with intestinal bacteria thus aggravated UC, while iron chelation therapy is effective in the treatment of colitis [41]. Since lipid peroxidation product such as MDA causes changes in the fluidity and permeability of cell membranes, ultimately leading to changes in cell structure and function [32], lipid peroxidation is considered another main molecular mechanism of toxicity process leading to cell death. Lipid peroxidation depends on the formation of free polyunsaturated fatty acids and the synthesis of free fatty acids requires the involvement of ACSL4 [42]. GPX4 and its cofactor GSH are the main pathways for the elimination of lipid peroxidation products which can reduce lipid hydroperoxides to nontoxic lipid alcohols, thereby limiting the propagation of lipid peroxidation within the membrane [43]. It has been found that lipid peroxidation occurs and ferroptosis is induced, when excess iron in the intestine produces ROS via the Fenton reaction which destroyed the intestinal epithelial cells and damaged the intestinal mucosal barrier resulting in UC [44, 45]. Therefore, in this study, the intestinal iron was used to respond to the level of iron metabolism, and MDA, GSH, GPX4, and ACSL4 were used to reflect the level of lipid peroxidation. In addition, significant downregulation and upregulation of ferroptosis-associated genes highlight the close relationship between ferroptosis and UC. Yang et al. found that the upregulation of NRF2 could inhibit ferroptosis and attenuate colitis-related mucosal damage and colonic inflammation [46]. In addition, Wang et al. clarified the vital role of GPX4 in negatively regulating ferroptosis in UC [36]. In this experiment, increased iron, MDA, and ACSL4 and decreased GPX4 and GSH in patients with UC also demonstrated the presence of ferroptosis, which is consistent with the conclusion of other studies [34, 35].

Researches on ERCs are now well mature, and it is well known that ERCs highly express MSC surface markers such as CD44, CD73, and CD105 and lowly express CD45, CD79a, and HLA-DR. In addition, studies demonstrated that ERCs can exert therapeutic effects through regulating immune response [47], promoting damage recovery [48], and differentiating into targeted cells [19, 49], and so on. Moreover, ERCs' clinical curative effects have been confirmed, Ichim et al. revealed that ERCs could improve heart function of patient suffering from dilated cardiomyopathy [50], and Xu et al. showed that ERC infusion could improve severe and critical COVID-19 [51]. ERC-exos are virtually unaffected by biological barriers [52] and safer to the host compared with ERCs [53], which could serve as a convincing novel type of cell-free treatment. Current researches indicated that MSC-exos act by following mechanisms. The wide repertoire of miRNAs in MSC-exos could provide a miRNA-based mechanism for the therapeutic effects of MSC-exos. Wang et al. revealed that exosomal miR-223 contributed to MSC-mediated cardioprotection in sepsis [54], and Xin et al. demonstrated that miR-17-92 in MSC-exos enhanced neurological [55]. Notably, lncRNA and circRNA also play an important role in the function of MSC-exos [56, 57]. In addition, proteins in MSC-exos have the potential to modulate the biological processes of diseases including myocardial ischemia/reperfusion injury [58], cystinosis [59], renal injury [60], and hyperoxic lung injury [61]. Apart from RNA species and protein, the effect of exosomal DNA cannot be ignored [62]. Kitai et al. confirmed that exosomes acted by packaging and transferring their mitochondrial DNA to targeted cells [63]. Ferroptosis is involved in the progression of numerous diseases, and it has been proved that MSC-exos could attenuate diseases through inhibiting ferroptosis. Studies have found that MSC-exos could deliver its miRNA and proteins that act on genes participating in iron metabolism and lipid peroxide process to regulate ferroptosis. Song et al. revealed that divalent metal transporter 1 is a target gene of miR-23a-3p carried by MSC-exos, and miR-23a-3p could suppress divalent metal transporter 1 expression to inhibit ferroptosis [64]. BECN1 is a biofactor of ferroptosis delivered by MSC-exo. Tan et al. proved that MSC-exos may promote xCT/GPX4-mediated activated hepatic stellate cells ferroptosis through the delivery of BECN1 [65]. Lin et al. also concluded that MSC-exos might alleviate ferroptosis via the CD44 and OTUB1-mediated stabilization of SLC7A11 [66]. Therefore, we speculated that ERC-exos regulates ferroptosis primarily through delivering its rich proteins and miRNA. In the coming period, we will further explore the role of proteins of ERC-exos in ferroptosis in UC. Studies have showed that ERC-exos can improve liver function [67], protect myocardial tissue [68], inhibit tumor cell growth [69], and improve the regenerative capacity of *β* islets [70]. To validate the role of ERC-exos on UC, the DSS-induced experimental colitis model was used, and pathology, DAI, and weight changes of mice were assessed. For further revealing the effect of ERC-exos on ferroptosis, in an vitro experiment, the erastin-induced group were compared with the ERC-exos-treated group. The results showed that the use of ERC-exos significantly downregulated ferroptosis in NCM460. Furthermore, the effects of ERC-exos on ferroptosis were confirmed in an *in vivo* model. Consistent with the advanced results, GPX4 expression and GSH synthesis were increased in the ERC-exos-treated group, while iron, MDA, and the expression of ACSL4 were decreased in the DSS-induced group, which suggested that ERC-exos can downregulated ferroptosis in colitis.

Programmed cell death such as apoptosis and pyroptosis are essential parts in the process of the disease. It has been proved that the inhibition of intestinal epithelial cell apoptosis and macrophage pyroptosis facilitate remission of colitis [71, 72]. Ferroptosis is a new form of programmed cell death characterized by increased iron and lipid peroxidation. In this study, we have confirmed that ferroptosis was involved in the pathogenesis of UC and ERC-exos did improve ferroptosis-related factors and thus alleviate the symptoms of UC. However, our study has some experimental limitations, the underlined specific mechanisms towards regulation of ferroptosis were not explored, and the comparison with ferroptosis inhibitors was not performed. Therefore, it is necessary to explore the detailed mechanisms of ERC-exos-mediated therapeutic effects in the future.

## 5. Conclusion

In conclusion, the results in this study have demonstrated that ferroptosis is involved in the pathogenesis of UC and ERC-exos can attenuate colitis in mice through downregulation of ferroptosis, which can provide a novel insight for the UC treatment.

## Data Availability

All data included in this manuscript can be available.

## Abbreviations

ACSL4: Acyl-CoA synthetase long-chain family member 4
BM-MSCs: Bone marrow cell derived exosomes
CCK-8: The Cell Counting Kit-8
DAI: The disease activity
DSS: Dextran sulfate sodium
ERCs: Endometrial regenerative cells
ERC-exos: Endometrial regenerative cell-derived exosomes
GPX4: Glutathione peroxidase 4
GSH: Glutathione
IBD: Inflammatory bowel disease
MDA: Malondialdehyde
MSCs: Mesenchymal stem cells
MSC-exos: Mesenchymal stem cell-derived exosomes
ROS: Reactive oxygen species
UC: Ulcerative colitis.
